# A state‐wide initiative to promote genetic testing in an underserved population

**DOI:** 10.1002/cam4.1100

**Published:** 2017-05-29

**Authors:** Meghan L. Underhill, Traci M. Blonquist, Karleen Habin, Debra Lundquist, Kristen Shannon, Kathryn Robinson, Mary‐Lou Woodford, Jean Boucher

**Affiliations:** ^1^ Dana‐Farber Cancer Institute 450 Brookline Ave LW522 Boston Massachusetts 02115; ^2^ Massachusetts General Hospital Boston MA USA; ^3^ Boston College Connell School of Nursing Cancer Resource Foundation Chestnut Hill MA USA; ^4^ School of Nursing Northeastern University Boston MA USA; ^5^ Cancer Resource Foundation Marlborough MA USA; ^6^ Graduate School of Nursing University of Massachusetts Medical School Worcester MA USA

**Keywords:** Cancer, disease susceptibility, genetic testing, healthcare disparity, nursing research

## Abstract

Genetic testing for cancer susceptibility has been widely studied and utilized clinically. Access to genetic services in research and practice is largely limited to well‐insured, Caucasian individuals. In 2009, the Cancer Resource Foundation (CRF) implemented the Genetic Information for Treatment Surveillance and Support (GIFTSS) program to cover the out‐of‐pocket expenses associated with cancer genetic testing, targeting high‐risk individuals with limited financial means and limited health insurance coverage. Here, we (i) describe the characteristics of participants in the Massachusetts (MA) GIFTSS program and (ii) evaluate mutations found in this diverse sample. A secondary retrospective data analysis was performed using de‐identified demographic data obtained from laboratory requisition forms and cancer genetic testing result information from the laboratory source. Eligible participants were those who utilized the MA GIFFTS program from 2009 through December of 2014. Data were summarized using descriptive measures of central tendency. Participants were residents of Massachusetts who had health insurance and had a reported income within 250–400% of the federal poverty level. Genetic testing results were categorized following clinical guidelines. Overall, 123 (13%) of participants tested positive for a mutation in a cancer susceptibility gene. For those with a cancer diagnosis, 65 (12%) were found to have a positive result and 20 (7%) had a variant of uncertain significance (VUS). For those unaffected patients, 58 (15%) had a positive result and 10 (3%) were found to have a VUS. The results from this study are useful in describing genetic testing outcomes in this high‐risk underserved community. Repeatedly, the literature reports that individuals from diverse or limited resource settings are less likely to access genetic testing. Continued research efforts should be devoted to promoting the access of genetic testing in the high‐risk, underserved community.

## Introduction

Results from cancer predisposition genetic testing are an important component of holistic cancer care and can inform treatment, diagnostic, surveillance, and preventative health recommendations 1, 2. Patient experience and outcomes of cancer predisposition genetic testing has been widely studied, but is largely limited to well‐insured, Caucasian populations with easy access to genetic counseling and testing services. Information pertaining to underserved communities is limited. Underserved communities include individuals with limited financial means, and low‐socioeconomic status, who may also be racial or ethnic minorities and face barriers to genetic testing services. Thus, underserved populations remain understudied.

Costs of the genetic test and limitations to health insurance coverage are barriers to genetic testing. Cragun et al. (2015) identified that having public health insurance, with an implied limitation to coverage was a barrier to utilizing genetic testing in young black women 3. Low household income 4, and/or low education 5 are also associated with poor uptake of genetic testing, with most data coming from *BRCA1/2* gene testing. Out‐of‐pocket costs associated with genetic testing even in the insured have also been identified as a barrier, regardless of race or ethnicity 6. Including individuals who are cared for in the community is a priority at the national level, though still equitable representation of diverse individuals has not yet been realized 7. Therefore, data available related to outcomes of genetic testing in diverse and unselected populations, are often those not included in historical clinical research, and is limited.

In 2009, the Cancer Resource Foundation (CRF), a 501(c)(3) program led by oncology and public health nurse leaders, initiated the Genetic Information for Treatment Screening and Surveillance (GIFTSS) voucher program in Massachusetts (MA) to support the underinsured who could not afford the out–of‐pocket expenses associated with genetic testing. Underinsured individuals were defined as those individuals who have health insurance that covers some health costs, but whose health insurance neglects to cover the entire costs of specific medical tests. This leaves these underinsured with either no access to the specific test or with a high out‐of‐pocket cost. At the time of inception of the program, the MA Healthcare Reform Act (HCRA), which was enacted in 2006, provided comprehensive health insurance to adults earning up to 150% of the federal poverty level. The MA HCRA did not include genetic testing as a covered service. Because individuals covered under the HCRA did have health insurance, participants in this program were not eligible for local or national financial relief programs. Thus, those individuals covered under HCRA had neither access to genetic testing as a covered benefit through their insurance nor the ability to seek outside relief programs.

The eligibility of individuals for the GIFTSS program met medical high risk as defined by the National Comprehensive Cancer Network (NCCN) 1, 2 if appropriate, and the referring clinician verified that the participant was appropriate for genetic testing. Referring clinicians were typically clinical genetic counselors, OB/GYN providers, and medical oncologists. Participants also met a financial need criterion and were considered eligible if they had a household income within or below 250–400% of the federal poverty level, and had health insurance that did not cover genetic testing. The criteria for financial inclusion was broadened from 250 to 400% to meet the needs of not only those whose insurance offered no coverage for genetic testing but also for those who did have some coverage but could not afford the out‐of‐pocket expenses.

The CRF relied on referring clinical experts (e.g., genetic counselors) to identify at‐risk individuals who needed referral to the GIFTTS resource. The patient and provider completed the application for voucher assistance. Once completed, the provider ordered genetic testing and CRF evaluated eligibility based on financial need. Once an application is submitted, a voucher to cover the out‐of‐pocket costs associated with obtaining genetic testing was provided. No direct clinical care, genetic counseling, or direct (cash) reimbursement to the patient was provided as part of GIFTSS, although advanced practice nurses with expertise in cancer and genetics were available to facilitate answering questions or identifying resources as needed. Previous research conducted with a subset of participants who took part in GIFTSS had indicated that the program included a diverse group of individuals 8. Data from our first study (*N* = 128) included participants from the same cohort and indicated that approximately half of the participants had a high school education or less, over half utilized state health insurance, and were from over 31 different ancestral backgrounds 8.

## Objectives

The objectives of this analysis were to: (i) describe the characteristics of participants from the MA GIFTSS program and (ii) evaluate the types of mutations found in this sample.

## Method

A secondary analysis of data collected from 2009 to 2014 was completed using de‐identified demographic and genetic testing results obtained from the laboratory source. Partners Healthcare Institutional Review Board reviewed the protocol and determined it exempt. All data were summarized using descriptive statistics (frequency and proportion). Age at the time of genetic testing and cancer diagnosis were reported using median and range. Individuals were categorized based on gender, ancestry (as classified by the referring provider), presence or absence of a cancer diagnosis, timing of genetic testing in relation to a cancer diagnosis (before a cancer diagnosis/unaffected, at the time of diagnosis, or after cancer diagnosis), genetic testing results, and presence or absence of a reported family history of cancer. Genetic testing results were defined by three categories 9: (i) Positive, where a deleterious gene mutation or likely deleterious mutation was identified, (ii) Uncertain, where a DNA variant of uncertain significance (VUS) or favor polymorphism (likely benign), was identified, and (iii) Negative, where no mutation was identified.

## Results

Genetic testing results were available for 927 of 928 participants who took part in the voucher program. Participant characteristics are detailed in Table 1. The median age at the time of genetic testing was 46 years (range 3–86 years). Ten individuals were tested under the age of 18 (range 3–17). All minors were tested for mutations in the *APC* gene due to risk for Familial Adenomatous Polyposis, which is clinically indicated as standard of care 2, 10.

**Table 1:** Patient characteristic and gene mutation summary for those tested

Participant characteristic	*N* (%)
*N*	927
Gender	
Female	839 (90)
Male	78 (8)
Unknown	10 (1)
Ancestry (may select more than 1)	
African	83 (9)
Asian	30 (3)
Central/Eastern Europe	86 (9)
Latin American/Caribbean	181 (19)
Native American	39 (4)
Neareast/Mideast	11 (1)
Western/Northern Europe	459 (49)
Unknown	140 (15)
Ashkenazi descent	32 (3)
Timing of genetic testing in relation to cancer diagnosis^a^	
Before cancer diagnosis (unaffected)	392 (42)
At same age as cancer diagnosis	227 (24)
At age after cancer diagnosis	281 (30)
Unknown	27 (3)
Age at genetic testing, median (range) [*n* = 913]	46 (3‐86)
Gene mutation^b^	151 (16)
Deleterious	118 (13)
Likely	5 (<1)
Uncertain	30 (3)
Family history based on presence or absence of cancer diagnosis	
With a cancer diagnosis	541 (58)
Family history of cancer	413 (76)^c^
No family history of cancer	127 (23)^c^
Not reported	1 (<1)^c^
Without a cancer diagnosis	386 (42)
Family history of cancer	378 (98)^c^
No family history of cancer	8 (2)^c^

a Based on age of genetic testing and age of cancer diagnosis.

b Four individuals had more than 1 mutation; two with a deleterious mutation and a VUS (variant of uncertain significance), and two individuals with 2 VUS.

c Percentage calculated from the number developing or not developing cancer.

There were 123 (13%) individuals with gene mutations identified that were considered positive.

The family history information of the participants can be found in Table 1. For those individuals who had a cancer diagnosis, 413 (76%) also reported a first‐degree relative (FDR) with cancer and for those who did not have a cancer diagnosis, 378 (98%) had a FDR with cancer. The age at time of first cancer diagnosis was known for 522 individuals. The median age at diagnosis was 44 years (range 16–78 years). The number of individuals with a cancer diagnosis who received genetic testing at the same time or after their cancer diagnosis was 508 (54%).

Participants underwent a variety of cancer genetic tests, including single‐site gene testing for known family mutations, multisite gene testing, multi‐gene panel, and large rearrangement testing. The genes analyzed for the cohort included: *BRCA1, BRCA2, MLH1, MSH2, MSH6, PMS2, EPCAM, APC, MYH, CDKN2A, CDK4, TP53, PTEN, STK11, CDH1, BMPR1A, SMAD4, PALB2, CHEK2, ATM, NBN, BARD1, BRIP1, RAD51C,* and *RAD51D*. Table 2 describes the name of the tests completed, the genes associated with that test, and the frequency with which the test was used in this sample. Table 3 describes the frequency of genetic tests completed and mutations detected.

**Table 2:** Frequency and type of tests ordered

Name of test	Genes included in test	*N*	%
Myrisk	BRCA1, BRCA2, MLH1, MSH2, MSH6, PMS2, EPCAM, APC, MYH, CDKN2A, CDK4, TP53, PTEN, STK11, CDH1, BMPR1A, SMAD4, PALB2, CHEK2, ATM, NBN, BARD1, BRIP1, RAD51C, RAD51D	18	2
BART	BRCA1, BRCA2	352	38
BRCA analysis	BRCA1, BRCA2	554	60
Single‐site BRCA1	BRCA1	59	6
Single‐site BRCA2	BRCA2	49	5
Multisite	BRCA1, BRCA2	24	3
APC	APC	19	2
Colaris	MLH1, MSH2, EPCAM, MSH6, PMS2, APC, MYH	62	7
Colaris AP	MLH1, MSH2, EPCAM, MSH6, PMS2, APC, MYH	64	7
PMS2	PMS2	59	6
MLH1 panel	MLH1	2	0
MSH2 panel	MSH2	3	0
MYH	MYH	24	3
EPCAM	EPCAM	2	0
Single‐site MLH2	MLH1	4	0
Single‐site MSH2	MSH2	13	1
Single‐site MSH6	MSH6	5	1

**Table 3:** Number of individuals tested for a gene mutation in each gene and the results of that testing

Gene tested	Associated cancers or cancer syndrome	No. tested	Result	No. with findings *N* (%)^a^, ^b^
No. of patients		927	‐	151 (16)
Deleterious/likely mutations				123 (13)
APC	Familial Adenomatous Polyposis (FAP)	94	APC – Deleterious	6 (6)
MYH	MYH‐associated polyposis (MAP)	89	Biallelic MYH	1 (1)
			Monoallelic MYH	5 (6)
BRCA1	Hereditary Breast and Ovarian Cancer Syndrome (HBOC)	745	BRCA1 – Deleterious	50 (7)
BRCA2	HBOC	735	BRCA2 – Deleterious	45 (6)
			BRCA2 – Likely	3 (<1)
MLH1	Lynch Syndrome (LS)	118	MLH1 – Deleterious	3 (2)
MSH2	LS	125	MSH2 – Deleterious	6 (5)
			MSH2 – Likely	2 (2)
MSH6	LS	117	MSH6 – Deleterious	2 (2)
VUS				30 (3)
APC	FAP	94	APC	4 (4)
ATM	Breast and pancreatic cancers	18	ATM	1 (6)
MYH	MAP	89	MYH	2 (2)
BRCA1	HBOC	745	BRCA1	3 (<1)
BRCA2	HBOC	735	BRCA2	10 (1)
BRIP1	Breast and ovarian cancers	18	BRIP	3 (17)
MSH2	LS	125	MSH2	1 (<1)
MSH6	LS	117	MSH6	5 (4)
PMS2	LS	118	PMS2	3 (2)

a Four individuals had more than 1 mutation; two with a deleterious mutation and a VUS, and two with 2 VUS.

b Percentage calculated from those tested.

The breakdown of mutations by cancer diagnosis is described in Table 4. Of those with a cancer diagnosis, 63 (12%) individuals reported more than one diagnosis of cancer. Sixty‐five (12%) were found to have a deleterious or likely deleterious mutation and 20 (7%) had a VUS. For unaffected participants, 10 (3%) were found to have a VUS and 58 (15%) had a deleterious/likely deleterious mutation and of which 46 (79%) of those were found through single‐site testing for a known genetic mutation within a family. Single‐site gene testing (15%) was performed on 140 individuals (15%), a positive gene mutation was identified in 62 (44%) participants. Of those who underwent single‐site testing, 108 individuals were unaffected.

**Table 4:** Number of mutations detected based on cancer diagnosis

	Total number of cancer diagnoses	Mutations identified	Number of deleterious	Number of likely	Number of VUS
Cancer diagnosis					
Breast^a^	400	BRCA‐1	18	–	3
		BRCA‐2	21	3	3
		BRIP1	–	–	1
Ovary	27	BRCA‐1	4	–	–
		BRCA2	1	–	–
Colorectal^a^	49	APC	1	–	–
		MLH1	2	–	–
		MYH	–	–	1
		Mono‐MYH	1	–	–
		MSH2	1	–	1
		MSH6	1	–	2
		PMS2	–	–	1
		BRIP1	–	–	1
Colon adenomas/polyps	60	APC	2	–	3
		MYH	–	–	2
		Bi‐MYH	1	–	–
		Mono‐MYH	2	–	–
		MSH2	2	2	–
		MSH6	–	–	2
		PMS2	–	–	2
		BRCA2	–	–	1
Desmoid tumors	1	–	–	–	–
Jejunum	1	MSH2	1	–	–
Pancreas	6	BRCA2	–	–	1
Prostate	3	MSH2	–	1	–
Endometrial/uterine	15	Mono‐MYH	1	–	–
		MSH2	–	1	–
Renal pelvis	2	MSH6	1	–	–
Fallopian tube	3	BRCA1	1	–	–
Cervix	5	MSH2	1	–	–
Melanoma	7	MLH1	1	–	–
Thyroid	4	–	–	–	–
Bladder	1	–	–	–	–
Leukemia	3	–	–	–	–
Sarcoma	1	BRCA1	1	–	–
Sebaceous	3	MSH2	2	–	–
Other	21	BRCA1	2	–	–
		Mono‐MYH	1	–	–
		MSH2	–	–	1
No cancer diagnosis	386				
		APC	4	–	1
		ATM	–	–	1
		BRCA1	26	–	–
		BRCA2	23	–	5
		BRIP1	–	–	1
		MLH1	1	–	–
		Mono‐MYH	2	–	–
		MSH6	–	–	2
		MSH2	2	–	–

a *N* = total number of identified mutations and includes individuals with more than 1 mutation; 63 individuals reported more than 1 cancer diagnosis (55 reported 2 and 8 reported 3), and therefore the individual participant's gene mutation may be represented within multiple cancer diagnoses.

## Discussion

Here, we report individual‐level genetic findings in an at‐risk cohort of program participants with low‐socioeconomic status in MA. Our findings demonstrate that genetic testing can provide valuable information within this community and to reach equitable genetics care, reducing barriers to accessing genetic testing should be addressed. The findings from this study should be interpreted within the socialecological context of the multiple systems that impact genetic testing uptake, including health system, policy, and community‐level factors 11. The unmet need filled by this voucher program highlights a gap in the current health care systems ability to meet the capacity of a socially and economically diverse community.

In our sample of individuals at high risk for having a genetic predisposition to cancer who had financial need for assistance to obtain genetic testing, 42% of participants were unaffected with cancer, and of those who were unaffected, 98% had a first‐degree relative (FDR) with cancer. Of those with a cancer diagnosis, the median age of onset was young, age 44. Therefore, our findings indicate that referral to GIFTSS was likely highly selective and that those individuals were likely appropriately referred, though this could not be confirmed. Our cohort's testing included genes other than *BRCA1* and *BRCA2*, a focus of most research in diverse groups 12, 13, 14, 15, 16, making our work novel to the current state of genetic testing in routine care. A clinically actionable genetic mutation was found in 13% of our sample, while 3% had a VUS, which is consistent of rates of mutations identified in other underserved populations 14, 17, 18. Therefore, in this sample, when financial barriers were removed, we see that this population had comparable rates of positive genetic testing results as reported in the literature.

Single‐site testing for a familial mutation was performed in 15% of our sample. In this subset of patients, 44% tested positive, and were given recommendations for medical management based on their results. For those individuals in the subset who did not have a familial mutation, the emotional and financial benefit of learning of these negative results and not needing additional medical intervention may be beneficial at both the individual and at the larger economic level.

In 2006, MA was the first state to enact the HCRA, followed by the National Affordable Care Act (ACA) signed into law 3/23/2010 with most provisions taking place on January 1, 2014. Although universal healthcare is mandatory in the state of MA, it seems clear that the state still has a significant population of underinsured individuals when it comes to genetic testing services. This study included over 900 participants who received services from 2009 to 2014. Updated data from 2016 indicates that over 1600 Massachusetts residents have now received services from the GIFTSS program, clearly demonstrating a need in our community. The ACA has a provision to offer “essential health benefits” for preventive and wellness services, guided by the US preventative Services Task Force. Genetic testing covered under this act includes testing for “BRCA‐related Cancer”. These benefits do not extend to those with a diagnosis of cancer or more rare cancer syndromes.

Additionally, laws surrounding health insurance, specifically for those with federal insurance, vary from state to state and often impact the underserved 6. Even with the ACA in place, certain private and public health plans do not cover services such as genetic testing. Some plans were grandfathered into the ACA and therefore, not required to meet current standards and recommendations of the United States Preventative Services Task Force (USPSTF) 19. Government insurance programs have limitations on who can be covered for genetic testing and when that genetic testing can occur. For example, Medicare enrollees unaffected by cancer do not meet criteria for coverage of genetic testing 6. Both the ACA and Medicare policy would have eliminated coverage for 386 individuals in our sample, 15% who had a clinically actionable gene mutation that may have direct relevance to cancer treatment and care for at‐risk relatives. Therefore, without assistance through the GIFTSS program, these at‐risk individuals and their families could have potentially missed the opportunity to know their cancer risk, which has individual, family, health care system, and societal implications.

Some private insurers place additional restrictions. For instance, genetic counseling by an approved genetic counselor is required prior to testing. This may limit access in rural or underserved communities where genetic counselors might not be available. Alternative models of providing cancer genetic counseling might be of benefit to at‐risk individuals, such as through telegenetic counseling or video counseling. In a randomized trial, telegenetic counseling has been shown to be noninferior to in‐person counseling, although it did lead to lower rates of genetic testing uptake 20. Utilizing a novel method of genetic counseling might help leverage resources to bring qualified professionals into the community. Other research indicates that telegenetics is considered convenient by participants 21 and more cost effective compared to in‐person counseling 22. Video counseling has also been shown to be feasible and acceptable to patients 23, 24. The importance of identifying new ways of providing genetic services to communities that serve individuals who are racially, ethnically, or socially diverse is exceedingly important given the lack of diversity in the current workforce of genetic counselors 25 and low rates of genetic testing referral in racial or ethnic minority groups 26. Therefore, to continue to reach underserved communities, genetic counselors and other genetics clinicians should utilize different approaches to make genetic counseling convenient and accessible.

Furthermore, the results of genetic testing may lead to additional treatment, diagnostic, or prevention recommendations. Co‐payments and deductibles for those services may prove to be prohibitive in pursuing treatment recommendations based on the results of the testing 27. The full magnitude of potential that comes from genetic testing can only be realized if at‐risk individuals, and their potentially at‐risk relatives, can access lifesaving early detection and prevention resources. Future research should expand upon this work to further evaluate how at‐risk individuals within this population are referred for and access follow‐up care. It remains to be seen if assisting high‐risk individuals in the MA community to access genetic testing can make a positive impact on cancer prevention and early detection in this population.

## Limitations

Our study has some limitations that should be noted. First, the accuracy of information entered on laboratory test requisition forms could not be verified. Although the test requisition forms ask for patient characteristics including family history information, it is unclear as to how deliberate these are completed by referring health care providers. Second, we were unable to capture additional socioeconomic information because this was a secondary data analysis. Further, sociodemographic data were limited to what was included on the laboratory case report forms. Therefore, data such as work status, marital status, and other sociodemographic characteristics were not included. Finally, we were unable to identify the number of unique families included in our sample because data were de‐identified and specific mutation information was not available. It is possible that multiple individuals from the same family may have been included which may inflate the mutation rate.

## Conclusion

The results from this study are useful in describing genetic testing outcomes in this high‐risk underserved community. Future work is needed to evaluate mutation rates in diverse samples to present a more inclusive description of the landscape of hereditary cancer risk in the general population. Continued research efforts should be devoted to promoting access to genetic testing in the high‐risk underserved community.

## Conflict of Interests

None declared.
